# Correction: Corneal epithelial remodeling induced by combined small incision lenticule extraction and accelerated corneal collagen crosslinking for myopia

**DOI:** 10.1371/journal.pone.0300941

**Published:** 2024-03-14

**Authors:** The PLOS ONE Staff

## Notice of republication

This article was republished on March 5, 2024, to correct an error in the title that was introduced during the typesetting process. The publisher apologizes for this error. Please download this article again to view the correct version. The originally published, uncorrected article and the republished, corrected articles are provided here for reference.

## Supporting information

S1 File Originally published, uncorrected article. (PDF)

S2 File Republished, corrected article. (PDF)
