# Supplementary material for: Correction: Corneal epithelial remodeling induced by combined small incision lenticule extraction and accelerated corneal collagen crosslinking for myopia
Source: PLoS One. 2024 Mar 14;19(3):e0300941. doi: 10.1371/journal.pone.0300941 (PMC10939202; doi:10.1371/journal.pone.0300941)
Supplement: S2 File — (PDF) [file pone.0300941.s002.pdf]

PLOS ONE

RESEARCH ARTICLE

Corneal epithelial remodeling induced by combined small incision lenticule extraction and accelerated corneal collagen crosslinking for myopia

Kook Young Kim[1], **Sinwoo Bae**[1], **Seongjun Lee**[1], **Yongwoo Lee**[2]*

1 Nuri Eye Hospital, Daejeon, Korea, **2** Department of Ophthalmology, Kangwon National University Hospital, Kangwon National University School of Medicine, Chuncheon, Korea

* gyska7@hanmail.net

Check for updates

OPEN ACCESS

Citation: Kim KY, Bae S, Lee S, Lee Y (2023) Corneal epithelial remodeling induced by combined small incision lenticule extraction and accelerated corneal collagen crosslinking for myopia. PLoS ONE 18(11): e0294121. https://doi.org/10.1371/journal.pone.0294121

Editor: Deepak Shukla, University of Illinois at Chicago, UNITED STATES

Received: June 23, 2023

Accepted: October 25, 2023

Published: November 8, 2023

Copyright: © 2023 Kim et al. This is an open access article distributed under the terms of the Creative Commons Attribution License, which permits unrestricted use, distribution, and reproduction in any medium, provided the original author and source are credited.

Data Availability Statement: The authors confirm that the data supporting the findings of this study are available within the article or its supplementary materials.

Funding: The author(s) received no specific funding for this work.

Competing interests: The authors have declared that no competing interests exist.

Abstract

Purpose

To evaluate the changes of the corneal epithelial thickness (ET) profile induced by combined small incision lenticule extraction and accelerated corneal collagen crosslinking (SMILE-xtra) for myopia compared with the standard small incision lenticule extraction (SMILE).

Setting

Nuri Eye Hospital, 61, Dunsan-ro, Seo-gu, Daejeon, 35233, Korea.

Design

Retrospective cross-sectional study.

Methods

Thirty-one myopic eye undergoing SMILE-xtra and control group of 36 myopic eyes undergoing SMILE were retrospectively analyzed. Spectral-domain optical coherence tomography (CIRRUS™ HD-OCT 5000, ZEISS, Dublin, CA) was used to measure corneal ET of 17 zones within the central 7-mm zone at preoperative, postoperative 1 month, 3 months and 6 months. Postoperative ET alterations were analyzed for correlation with treatment parameters.

Results

There was no difference in preoperative mean age, postoperative MRSE, visual acuity, and ablation depth between the two groups, and there was a significant difference in preoperative central corneal thickness. Both groups showed the greatest increase in corneal ET in the paracentral area on the inferotemporal area, respectively, for 6 months. The preoperative MRSE and the ablation depth showed significant correlation with the postoperative epithelial thickening in mid-peripheral sectors in both groups, and significant negative correlations in paracentral sectors only in SMILE-xtra group.

Conclusions

It is significant as the first study to compare corneal epithelial remodeling between SMILE and SMILE with accelerated corneal collagen crosslinking. The SMILE-xtra with the relatively large corneal ablation did not show a significant difference in the pattern of corneal epithelial remodeling compared to the SMILE group.

Introduction

The corneal epithelium is an anterior layer of the cornea that contributes to maintain the integrity of the ocular surface and the refractive power of the eye. Corneal epithelium plays a dynamic role in establishing corneal regularity that changes its thickness against stromal irregularities after corneal refraction surgery, contributing to a smooth ocular surface and improving vision [1, 2].

Corneal collagen cross-linking (CXL) utilizes photosensitizer (riboflavin) and ultraviolet-A light (UVA) to ultimately slow or prevent keratoconus progression by creating strong covalent bonds within the corneal stromal collagen strand, increasing stiffness. The simultaneous combination of prophylactic CXL and refractive cornea surgery improves the biomechanical stability of the post-operative cornea and potentially prevents iatrogenic ectasia [3]. With the development of prophylactic CXL, the indications for SMILE (small incision lenticule extraction) could be expanded even in high myopia or patient with ectasia risk factor [3, 4].

Many previous studies [5–7] have reported increased epithelial thickness (ET) following corneal refraction surgery, similarly, there are several studies [8–14] on corneal epithelial changes after SMILE. However, there have been no studies evaluated corneal epithelial remodeling patterns following the combined SMILE and prophylactic CXL (SMILE-xtra). This study compared corneal ET changes after SMILE and SMILE-xtra using spectral-domain optical coherence tomography (OCT).

Methods

Patients

We retrospectively reviewed the medical records of patients who had undergone SMILE and SMILE-xtra at Nuri Eye Hospital from January 2019 to October 2021. Data was accessed after IRB approval in December 2022. The study protocol was approved by the Institutional Review Board (IRB number: KNUH-2022-11-013) at Kangwon national university hospital, Chuncheon-si, Korea, and the study was conducted in accordance with the tenets of the Declaration of Helsinki. This study was a retrospective study with a level below the minimum risk and consent waiver was granted by the Institutional Review Board.

After the ophthalmologist determined if they were suitable for surgery, they underwent SMILE surgery following informed consent. The inclusion criteria were (1) bilateral myopia or myopia with astigmatism, (2) age older than 18 years and less than 45 years, (3) stable refraction error for at least 1 year, that is, a change $\leq$ of 0.50 diopters (D) in the spherical and cylindrical refraction (4) presence of myopia in manifest refraction spherical equivalent (MRSE) between − 1.00 D and − 8.00 D, (5) presence of astigmatism between 0.00 D and– 4.00 D, and (6) best preoperative corrected distance visual acuity of 0.8 (decimal value) or better in each eye. Soft contact lens wearers were instructed not to use them at least 15 days prior to surgery. The exclusion criteria were as follows: the use of hard contact lenses, a central corneal

thickness of less than 480 μm, a calculated postoperative residual stromal bed of less than 250 μm, and the presence of other ocular pathologic conditions such as corneal dystrophy, keratoconus, corneal opacity, or a history of previous ocular surgery.

Based on pre-operative examination, risk factors for corneal ectasis were assessed using the scoring system proposed by Randleman et al. [15]. Based on corneal topography, age, central corneal thickness, and expected postoperative remnant corneal stromal thickness, a score of 4 or more was classified as a high-risk group, a score of 3 was a moderate risk group, and a score of 2 or less was a low-risk group. Since the above classification was made based on LASIK surgery, the thickness of the corneal remnant matrix was calculated assuming that the cap thickness of SMILE was 120 μm. Eyes with moderate and high risk were selected for SMILE-xtra.

SMILE procedure

SMILE was performed using a VisuMax 500-kHz femtosecond laser (Carl Zeiss Meditec AG, Jena, Germany). The pulses of the laser were applied with a pulse energy of approximately 120 nJ. The spot distance of each laser spot was 4.0 μm. A 2 mm incision was made on the 145˚ meridian, and the upper and lower edges of the lenticule were delineated so that the tissue planes were clearly defined. The upper interface was separated, and the lower layer was dissected. When the two layers were separated, the lenticle was removed from the cornea. The diameter of the cap was 7.5mm and the optical zone diameter was 6.5mm. The intended cap thickness was 120 μm. CXL was performed as follows in SMILE-xtra. After lenticule extraction, Vibex Rapid ™ (Avedro, Inc., Waltham, MA, USA) containing 0.25% saline-diluted riboflavin mixed with a balanced salt solution was injected into the intrastromal pocket [16]. The corneal stromal bed was soaked with the solution for 60 seconds, followed by cleaning the interface with a saline solution. The surface was irradiated with 45 mW/cm^2 ultraviolet light of 375 nm using the KXL SystemⓇ (Avedro, MA, USA) for 75 seconds with a total energy of 3.4 J/cm2 and a diameter area treatment of 9.00 mm. [16] (Table 1). Postoperative medications included topical Levofloxacin 0.5% (CravitⓇ ophthalmic solution 0.5%; Santen Pharmaceutical Co., Ltd., Osaka, Japan) 4 times for 7 days, Loteprednol etabonate 0.5% (LoteproⓇ, Hanlim, Korea) in tapering dosages for 4 weeks, and artificial tear 4–6 times for 4 weeks or more.

Table 1. Prophylactic corneal cross-linking methods used during small lenticule extraction (SMILE) surgery in this study.

Parameter	Variable
Treatment target	Prophylaxis
Fluence (total) (J/cm^2)	3.4
Soak time (s)	60
Intensity (mW)	45
Epithelium status	On (intrastromal soaking)
Chromophore	Riboflavin (Vibex Rapid ™)
Chromophore carrier	Hydroxypropyl methylcellulose (HPMC)
Chromophore osmolarity	Iso-osmolar
Chromophore concentration	0.25%
Light source	KXL systemⓇ (Avedro)
Irradiation mode (interval)	Continuous
Protocol modifications	None
Protocol abbreviation in manuscript	SMILE-xtra

https://doi.org/10.1371/journal.pone.0294121.t001

Measurement of corneal epithelial thickness

The examination was performed by an one expert optometrist, and it included uncorrected and corrected distance visual acuity (UDVA and CDVA, decimal value), manifest refraction with and without cycloplegia by the fogging method of refraction. Astigmatism was assessed by the Jackson cross-cylinder technique. Corneal pachymetry, keratometry, and tomography patterns were measured with the Pentacam® AXL (Oculus Optikgeräte GmbH, Wetzlar, Germany).

The epithelial thickness (ET) and retinal optical coherence tomography were measured with spectral-domain Cirrus 5000 HD-OCT (Carl Zeiss Meditec, Germany). Corneal ET profiles of the central 7 mm zone were acquired preoperatively and at all follow-up measurements. Since the ET measurement in this study included the pre-cornel tear film, it was measured 1 minutes after instillation of a drop of artificial tear eyedrop (0.5% carboxymethylcellulose, Refresh, Allergan) before measurement [17]. In addition, topical eye drops were not permitted for a period of 2 hours prior to scanning to minimize their effect on tear film thickness. All OCT examinations were scheduled between the hours of 0900 and 1300 and were conducted by an trained optometrist to reduce the influence of diurnal and operator-related changes in ET measurements [18]. Two consecutive acquisitions were made for each patient to ensure the validity of the data, and the average value was used for the analysis. All OCT images were exported and processed using the Cirrus HD-OCT visualization software that provides an average automated ET of three ring-shaped concentric zones centered on the center of the cornea. The epithelial thickness maps were divided into a total of 17 sectors: a central 2 mm diameter zone, eight paracentral sectors within an annulus between the 2–5 mm diameter ring area, and eight mid-peripheral sectors within an annulus between the 5–7 mm diameter ring area (Fig 1).

Statistical analysis

The sample size was determined to have a power of at least 80% based on existing published results using MedCalc software (version 22.013, MedCalc Software Ltd., Ostende, Belgium) [8–14]. Data were analyzed using SPSS, version 24.0 (IBM Corporation, Armonk, NY, USA),

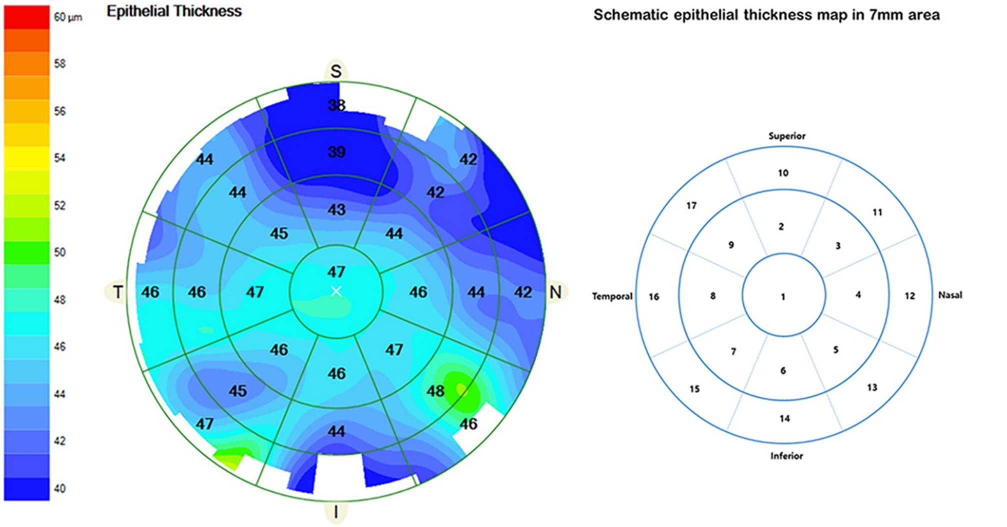

Fig 1. Example showing the measurements zones (sectors) with the spectral-domain Cirrus 5000 HD-OCT (Carl Zeiss Meditec, Germany).

https://doi.org/10.1371/journal.pone.0294121.g001

for Windows (Corporation, Redmond, WA, USA). The Shapiro–Wilk test was used to evaluate the normality of numerical data. The student's t-test was used if variables were normally distributed, whereas the Mann-Whitney U test was used if one or more variables were not normally distributed for analysis of the difference between the two groups. Continuous variables were compared using a two-way repeated measures ANOVA with the post hoc Bonferroni test, with follow-up time as a within-subjects factor (preoperative, 1 month, 3 months, and 6 months), and CXL treatment as a between-subjects factor (SMILE and SMILE-xtra). Spearman's coefficient was used to determine the association between the ET changes of different zones and treatment parameters. Statistical significance was set at $p < 0.05$.

Results

In this study, we enrolled a total of 67 eyes (36 eyes of SMILE, 31 eyes of SMILE-xtra) of 67 patients, and only one eye from each participant was randomly selected for the study. There was a significant difference of preoperative central corneal thickness, residual stromal thickness (RST) between two groups even though the mean age, preoperative manifest refraction spherical equivalent (MRSE), ablation depth, axial length and optical zone diameter (6.5 mm) were matched between the two groups (Table 2). There was no significant difference in the mean postoperative refractive error at 6 months (SMILE: -0.09 ± 0.11 D, SMILE-xtra: -0.14 ± 0.37 D, p = 0.812). The achieved spherical equivalent refraction versus attempted spherical equivalent refraction is presented in Fig 2. (R^2 = 0.995 in SMILE group, R^2 = 0.995 in SMILE-xtra group).

Table 3 showed the changes in corneal ET in 17 sectors. In all sectors, it showed an increasing pattern up to 3 months, and showed a stabilizing pattern between 3 and 6 months. There was a significant difference only in sector 16 (p = 0.029) at the first month by each follow-up period, and no significant difference was shown in other sectors. There was no statistically

Table 2. Demographic data.

	SMILE 36 eyes (Mean ± SD)	SMILE-xtra 31 eyes (Mean ± SD)	p-value
Age (years)	24.81 ± 3.83	23.39 ± 2.19	0.357*
Sphere (diopter)	−4.15 ± 1.14	−4.43 ± 1.56	0.203*
Cylinder (diopter)	−1.13 ± 0.89	−1.78 ± 0.79	0.001*
MRSE (diopter)	−4.72 ± 1.33	−5.35 ± 1.71	0.099**
Op. input MRSE (diopter)	−5.36 ± 1.56	−6.07 ± 1.94	0.103**
Mean K (diopter)	43.33 ± 1.26	43.31± 1.20	0.960**
Postoperative UCVA (decimal value)	1.086 ± 0.11	1.032 ± 0.07	0.722**
Postoperative MRSE	-0.09 ± 0.11	−0.14 ± 0.37	0.812**
Central corneal thickness (μm)	538.31 ± 31.03	507.55 ± 27.91	<0.001*
Ablation depth (μm)	97.61 ± 23.55	107.00 ± 24.09	0.113**
Residual stromal thickness (μm)	330.97 ± 32.42	291.19 ± 33.97	<0.001*
Ectasia Risk Score System	2.42 ± 1.32	4.87 ± 1.50	<0.001*
Percentage of tissue altered (PTA)	0.38	0.42	<0.001*
Axial length (mm)	25.58 ± 0.91	25.90 ± 0.99	0.178**

Abbreviations: MRSE, manifest refraction spherical error; UCVA, uncorrected visual acuity

*Mann-Whitney U test

**student-T test

https://doi.org/10.1371/journal.pone.0294121.t002

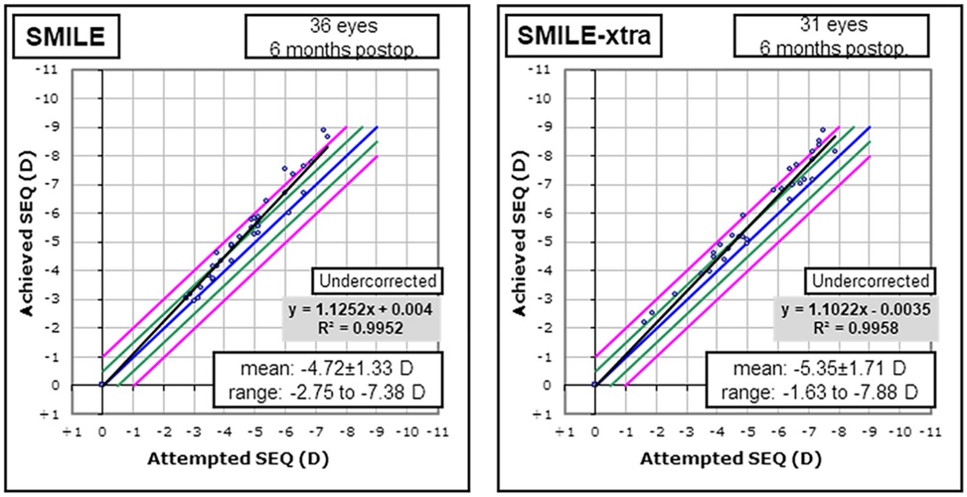

Fig 2. Predictability of spherical equivalent correction at 6 months, in SMILE group and SMILE-xtra group. Both groups showed a high level of refractive predictability.

https://doi.org/10.1371/journal.pone.0294121.g002

significant difference of the corneal ET in each area according to the follow-up time between the two groups in all sectors (two way-RM ANOVA, $p > 0.05$ in all sectors).

Fig 3 shows the sequential changes of average ET in 17 sections before and after SMILE and SMILE-xtra. During the 3-month follow-up, there was an increase in ET in all 17 areas, especially in the inferotemporal sectors than in others. This tendency was more pronounced in the SMILE-xtra group. Between 3 and 6 months, the epithelial changes were insignificant and maintained in both groups. Both groups showed the greatest increase in corneal ET in the paracentral area on the inferotemporal area, and the central sector showed a change of 4.15 μm (SMILE) and 3.90 μm (SMILE-xtra), respectively, for 6 months (Fig 4).

The longitudinal ET changes of annular zones are shown in Fig 5. The central and paracentral annular zone showed significant epithelial increase at each follow-up period up to 3 months in the SMILE group. However, in the SMILE-xtra group, the overall tendency of the increasing ET was similar to that of the SMILE group, but there was no statistical significance in the central area in the first 1 month, and significant ET increase was seen between 1 and 3 months. There was no statistical significance in any zone between 3 and 6 months.

Table 4 shows the correlation between ET change and age, preoperative MRSE, ablation depth, RST. There was no correlation between age and the postoperative ET change for 6 months in all annular zones. The preoperative MRSE showed significant negative correlation with the postoperative epithelial thickening in central 2 mm and mid-peripheral sectors in both groups, and significant negative correlations in paracentral sectors only in SMILE-xtra group. The ablation depth showed significant positive correlation with the postoperative epithelial thickening in mid-peripheral sectors in both groups, and significant positive correlations in paracentral sectors only in SMILE-xtra group. RST showed a significant negative correlation with corneal epithelial thickening in all areas only in the SMILE-xtra group.

Discussion

Corneal laser refractive surgery alters the anterior corneal contour that induces corneal epithelial remodeling to compensate for the change in corneal stroma. This compensatory change has been also described in myopic [19] and hyperopic [20] laser in situ keratomileusis (LASIK), photorefractive keratectomy (PRK) [21] before SMILE. In particular, the corneal

Table 3. Analysis of changes in corneal epithelial thickness between two groups in 17 sectors during the 6-month follow-up period.

		Preoperative (μm)	1 month (μm)	3 months (μm)	6 months (μm)	Time × subgroup		
						df	F	p-value+
1	SMILE	48.47 ± 2.97	50.17 ± 3.31	52.64 ± 3.75	53.14 ± 3.26	2.318	0.616	0.565
	SMILE-xtra	48.00 ± 2.02	48.94 ± 3.15	51.97 ± 3.58	51.90 ± 3.66			
	p-value*	0.394	0.176	0.441	0.247			
2	SMILE	45.86 ± 3.77	47.22 ± 3.60	50.17 ± 4.19	49.92 ± 4.11	2.113	1.148	0.322
	SMILE-xtra	45.68 ± 1.94	46.23 ± 2.29	48.74 ± 2.66	49.10 ± 3.07			
	p-value*	0.980	0.290	0.110	0.544			
3	SMILE	46.11 ± 3.66	47.47 ± 3.20	49.86 ± 3.36	49.72 ± 3.26	2.340	0.593	0.580
	SMILE-xtra	46.35 ± 2.01	46.87 ± 2.66	49.45 ± 2.99	49.48 ± 2.77			
	p-value*	0.509	0.330	0.600	0.899			
4	SMILE	47.36 ± 3.23	48.47 ± 3.17	51.19 ± 3.68	51.03 ± 3.78	2.203	0.059	0.955
	SMILE-xtra	47.55 ± 2.11	48.68 ± 2.48	51.23 ± 3.13	51.00 ± 2.53			
	p-value*	0.496	0.564	0.899	0.835			
5	SMILE	48.67 ± 3.42	50.50 ± 3.42	53.03 ± 3.11	53.06 ± 2.95	2.364	0.141	0.900
	SMILE-xtra	48.23 ± 2.36	50.10 ± 2.44	52.29 ± 3.15	52.32 ± 2.60			
	p-value*	0.551	0.737	0.346	0.443			
6	SMILE	48.92 ± 3.06	51.94 ± 3.14	54.58 ± 3.60	54.06 ± 2.96	2.634	0.342	0.769
	SMILE-xtra	48.00 ± 2.19	50.45 ± 2.71	53.10 ± 3.83	53.00 ± 3.52			
	p-value*	0.551	0.055	0.146	0.320			
7	SMILE	48.33 ± 3.61	52.39 ± 3.85	54.64 ± 3.60	54.06 ± 3.13	2.572	1.904	0.140
	SMILE-xtra	46.61 ± 2.09	51.68 ± 3.33	54.58 ± 4.15	54.00 ± 4.05			
	p-value*	0.053	0.452	0.965	0.975			
8	SMILE	47.14 ± 3.32	50.97 ± 3.73	53.53 ±3.20	53.33 ± 3.21	2.217	0.444	0.663
	SMILE-xtra	46.84 ± 2.04	51.13 ± 2.95	53.94 ± 3.88	53.81 ± 3.81			
	p-value*	0.685	0.523	0.738	0.587			
9	SMILE	46.50 ± 3.53	49.14 ± 4.23	51.19 ± 3.80	51.14 ± 3.68	2.212	0.280	0.778
	SMILE-xtra	46.16 ± 1.83	48.13 ± 2.62	50.48 ± 2.89	50.61 ± 3.23			
	p-value*	0.716	0.359	0.331	0.663			
10	SMILE	43.69 ± 3.38	43.25 ± 4.78	45.94 ± 5.26	45.58 ± 4.85	2.575	1.686	0.179
	SMILE-xtra	44.45 ± 2.55	43.94 ± 2.76	45.00 ± 2.48	45.81 ± 3.01			
	p-value*	0.140	0.222	0.709	0.686			
11	SMILE	45.00 ± 3.71	45.72 ± 4.05	47.17 ± 4.44	47.22 ± 3.88	2.403	0.186	0.867
	SMILE-xtra	45.77 ± 2.22	46.03 ± 3.18	47.58 ± 3.18	48.00 ± 2.93			
	p-value*	0.114	0.487	0.699	0.383			
12	SMILE	46.81 ± 3.42	47.67 ± 4.04	50.14 ± 3.98	49.94 ± 3.85	2.388	0.596	0.581
	SMILE-xtra	47.32 ± 2.26	48.90 ± 3.08	50.94 ± 4.07	50.16 ± 3.46			
	p-value*	0.240	0.163	0.390	0.503			
13	SMILE	48.00 ± 3.34	48.67 ± 4.01	50.25 ± 3.91	49.97 ± 3.31	3.000	0.327	0.806
	SMILE-xtra	48.16 ± 2.25	48.77 ± 3.27	50.94 ± 4.07	49.42 ± 3.33			
	p-value*	0.633	0.569	0.681	0.444			
14	SMILE	48.08 ± 4.01	48.50 ± 3.43	49.97 ± 4.61	50.33 ± 4.11	2.658	0.970	0.400
	SMILE-xtra	46.90 ± 2.87	47.87 ± 3.00	50.13 ± 4.80	49.16 ± 3.61			
	p-value*	0.141	0.565	0.870	0.322			
15	SMILE	46.97 ± 4.13	48.64 ± 4.35	50.47 ± 4.73	50.14 ± 4.11	2.412	0.934	0.410
	SMILE-xtra	46.03 ± 2.43	49.13 ± 4.43	50.55 ± 3.81	50.00 ± 4.18			
	p-value*	0.297	0.496	0.995	0.618			

(*Continued*)

Table 3. (Continued)

		Preoperative (μm)	1 month (μm)	3 months (μm)	6 months (μm)	Time × subgroup		
						df	F	p-value+
16	SMILE	45.86 ± 3.53	47.67 ± 4.83	49.75 ± 4.14	49.92 ± 3.52	2.536	1.399	0.248
	SMILE-xtra	46.35 ± 2.33	49.52 ± 3.44	51.77 ± 4.67	51.00 ± 4.07			
	p-value*	0.234	0.029	0.083	0.096			
17	SMILE	44.44 ± 3.77	45.31 ± 5.39	47.25 ± 4.40	47.08 ± 4.02	2.395	0.507	0.636
	SMILE-xtra	45.55 ± 3.20	45.58 ± 2.19	47.32 ± 3.09	47.58 ± 3.03			
	p-value*	0.186	0.191	0.960	0.672			

*Mann Whitney U test.

+ Two-way repeated ANOVA test.

https://doi.org/10.1371/journal.pone.0294121.t003

epithelial hyperplasia after corneal laser refractive surgery is related to myopic regression, so it is important to know the pattern of epithelial changes in predicting clinical outcomes [22, 23].

The corneal epithelial remodeling induced by SMILE has been reported in previous OCT based studies (Table 5). In this study, we used the Cirrus HD-OCT 5000 evaluate corneal epithelial remodeling of 7 mm circular zone over 6 months after SMILE and SMILE-xtra. This device has been demonstrated to produce excellent repeatability and reproducibility in normal [24] and keratoconus [25]. This study is meaningful that it is the first study to evaluate the change of the corneal epithelium in SMILE combined with corneal collagen cross-linking using this device.

Prophylactic CXL with corneal refractive surgery has been shown to enhance biomechanical strength and stability and prevent the risk of postoperative corneal ectasis. Previous studies [26, 27] have shown cytotoxic effects of riboflavin-UVA on corneal cells, including limbal epithelial stem cells and delayed corneal epithelial healing in patients following CXL [28].

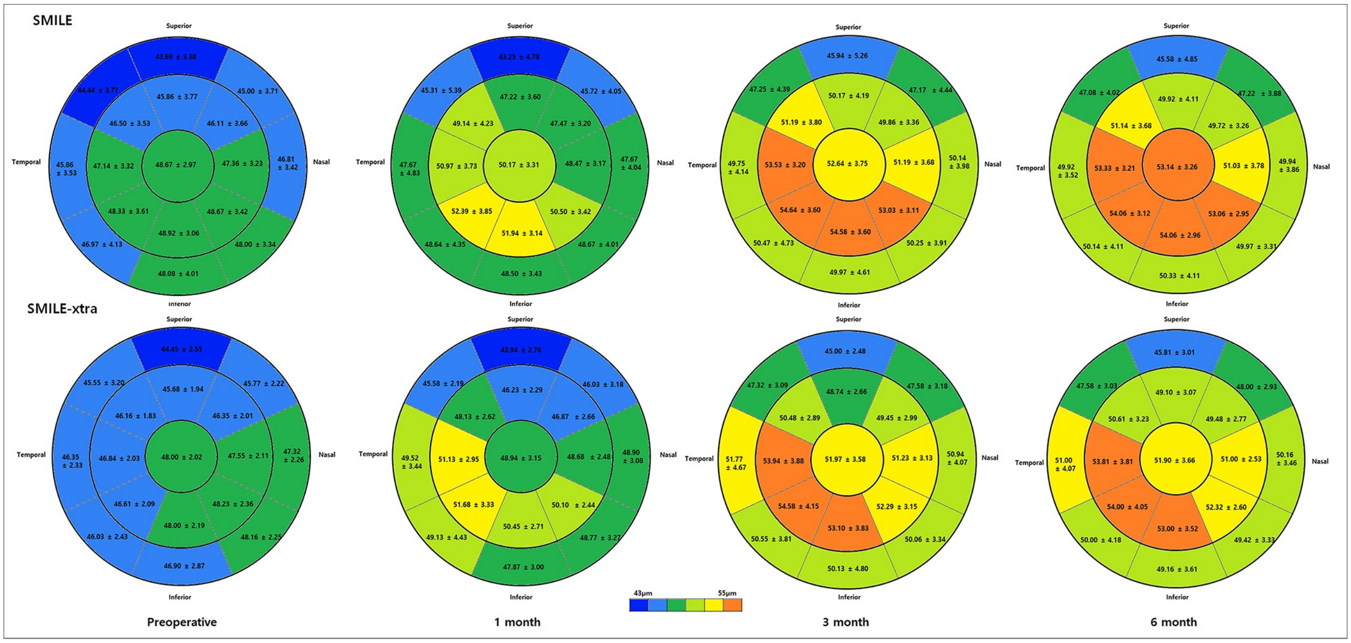

Fig 3. Mean sectoral ET at baseline and at 1, 3, and 6 months after surgery.

https://doi.org/10.1371/journal.pone.0294121.g003

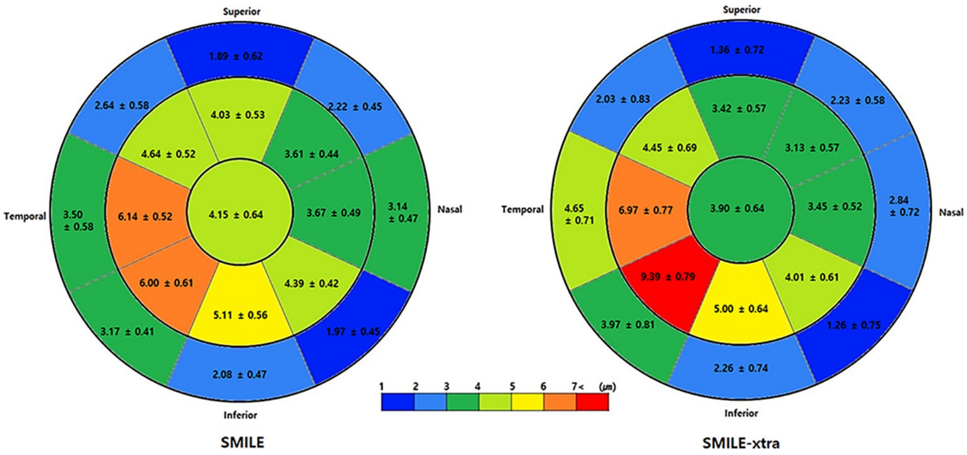

Fig 4. Epithelial thickness profile map: Absolute difference between 6 months postoperatively and baseline (mean ± SE).

https://doi.org/10.1371/journal.pone.0294121.g004

However, compensatory corneal epithelial changes were also found in the SMILE-xtra group, and it was found that the combination of accelerated CXL did not significantly affect corneal epithelial remodeling in this study. Rather, epithelial changes were more prominent in the SMILE-xtra group with a relatively large ablation depth. Unlike PRK or conventional CXL, corneal de-epithelization is not performed during SMILE-xtra surgery and the effect of riboflavin-UVA in the femtosecond laser created stromal pocket is relatively limited. Therefore, there is less possibility of affecting the corneal limbal epithelial stem cells, and it is thought that it will act more on the stromal side to induce effective collagen cross-linking [29].

In this study, the mean ET of the central 2 mm increased by 4.15 μm of SMILE group and 3.90 μm of SMILE-xtra group in six months postoperatively, which was relatively small compared to previous studies [9–11]. The discrepancy may be due to the different measuring instruments [30] and the different preoperative SE and race [31] of the participants. In the paracentral zone, our results demonstrated that epithelial hyperplasia is more pronounced in the temporal zone than in the nasal zone. This asymmetric profile is similar to previous reports [9, 11, 13, 14]. Ivarsen and Hjortdal [32] reported an abrupt stromal change with increased corneal ET corresponding to the edge of the removed lenticule at the astigmatism axis. The ablation depth is significantly different between preoperative flat and steep axis in eyes of high astigmatism, with a higher ablation depth applied to flat axis [33]. Hence, the discrepancy between the two meridional ablation depths may lead to different amounts of corneal epithelial hyperplasia, and more significant corneal epithelial hyperplasia may occur at the preoperative

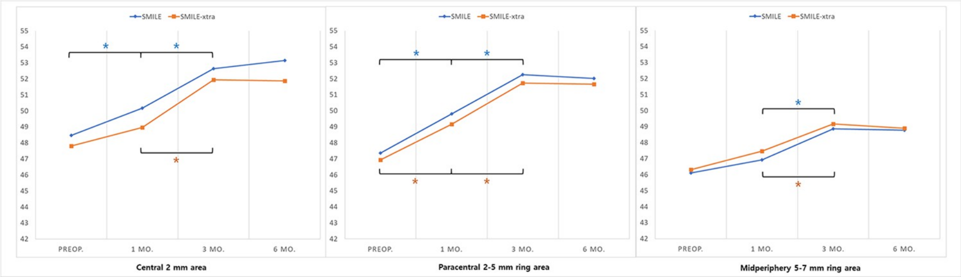

Fig 5. Preoperative to six-month postoperative epithelial thickness changes, in the central (2 mm), paracentral (2 to 5 mm), and midperipheral annular zones (5 to 7 mm).

https://doi.org/10.1371/journal.pone.0294121.g005

Table 4. Spearman correlation between change in corneal epithelial thickness and various factors.

	SMILE		SMILE-xtra	
	R	P	R	p
Age				
2 mm central area	0.223	0.191	–0.201	0.277
2–5 mm annular area	0.073	0.671	–0.136	0.465
5–7 mm annular area	-0.314	0.062	–0.059	0.752
Preoperative MRSE				
2 mm central area	–0.354	0.034	–0.401	0.025
2–5 mm annular area	–0.035	0.837	–0.538	0.002
5–7 mm annular area	–0.349	0.037	–0.453	0.011
Ablation depth				
2 mm central area	0.241	0.157	0.161	0.386
2–5 mm annular area	–0.035	0.838	0.363	0.045
5–7 mm annular area	0.400	0.016	0.481	0.006
RST				
2 mm central area	–0.112	0.515	–0.574	0.001
2–5 mm annular area	0.169	0.324	–0.763	<0.001
5–7 mm annular area	–0.026	0.878	–0.589	<0.001

https://doi.org/10.1371/journal.pone.0294121.t004

flat axis. The epithelial hyperplasia pattern response to corneal refractive surgery may be partially due to the mechanical influence of the upper eyelid tarsal during blinking. The Asian eyelid has a narrower palpebral opening, a larger eyelid volume and therefore a higher eyelid pressure. Previous research has suggested that there is a relationship between eye tension and changes in corneal topography, including induced astigmatism [34]. The corneal epithelial hyperplasia is more likely in the inferior portion of the cornea, which is easily in contact with the tear film, and can cause the vertical asymmetry of ET [35]. It is not clear why the corneal epithelial thickening at mid-peripheral area is greater than the central area changes after SMILE. For the above reasons, it is thought that a specific pattern occurs in the modeling of the corneal epithelium after SMILE and SMILE-xtra.

In this study, there was no significant correlation between age and corneal epithelial hyperplasia. This is a different result from previous study [9] and is considered to be a selection bias due to a relatively smaller age distribution (19 to 35 years) than previous study (25 to 45 years) and a different refraction error. The central and mid-peripheral 5–7 mm annular area showed a significant negative correlation with MRSE similar to previous studies [11, 14]. Ablation depth was relatively large in the SMILE-xtra group, but not statistically significant. Corneal epithelial hyperplasia showed a statistically significant correlation with ablation depth, especially in the paracentral areas (2-5mm and 5-7mm areas). The SMILE group with a relatively thick enough RST compared to the SMILE-xtra group showed no significant correlation with corneal epithelial hyperplasia, and the SMILE-xtra group with a relatively thin RST showed a significant negative correlation. It can be seen that the corneal epithelial hyperplasia is a compensatory growth in the ablated cornea, and more significant corneal epithelial hyperplasia is shown with a more corneal stromal ablation depth.

This study had some limitations. First, it had a retrospective design and a smaller sample size. Therefore, there might have been a selection bias in retrospectively comparing the SMILE-xtra group with from the SMILE group. Since corneal collagen cross-linking was additionally performed when the patient had a risk factor for corneal ectasia, there may be selection bias in the choice of surgical method. However, we tried to minimize the bias by comparing

Table 5. Previous studies of corneal epithelial remodeling after SMILE using OCT.

Eyes	Age	Follow-up period	Refractive error (D)	Mean ablation depth (μm)	OCT	Main finding
46 eyes[9]	33 ± 6	6 months	−4.78 ± 1.75 (-8.5 to -2.0)	94.3 ± 24.3 (5 to 143)	RS 3000 Advance (Nidek Co., Ltd., Gamagori, Japan)<br>– 5 mm zone	-Epithelial thickening of approximately 10% is observed during the first 6 postoperative months.<br>-The epithelial remodeling stabilized after 3 months and its extent is strongly determined by the amount of surgically induced refractive correction.<br>-The compensatory potential of the corneal epithelium decreases with increasing age.
100 eyes[10]	24.4 ± 2.8	3 months	−4.52 ± 2.18 (−1.25 to −9.75)	n	RTVue-OCT (Optovue, Inc., Fremont, CA)<br>– 6 mm area	-Most pronounced in the central (5.1 ± 2.2 μm) and superior zones (3.9 ± 2.1 μm) with no significant changes in the remaining zones<br>-A positive correlation between the degree of myopia corrected and the postoperative epithelial thickening was observed in the central ($r2 = 0.723$, $P < .001$) and superior ($r2 = 0.585$, $P < .001$) zones
113 eyes[11]	26.15 ± 5.53	3 months	-4.23 ± 1.27 (-2.00 to -7.00)	89.23 ± 21.37 (51 to 127)	RTVue-OCT (Optovue, Inc., Fremont, CA)<br>- 6 mm area	-Mid-peripheral epithelial thickening is greater than the central changes following SMILE.<br>-It is interesting to note that the increase in central epithelial thickness is smaller in the SMILE group despite the removal of a larger amount of stromal tissue compared with LASIK.<br>-Epithelial thickness changes are less marked centrally but increased radially (centrifugally) toward the mid-periphery.
42 eyes[12]	29.6 ± 6	24 months	-5.61 ± 2.02 (-2.12 to -10.00)	n	RTVue 100 (Optovue Inc, Fremont, CA)<br>- 6 mm area	-A larger increase in epithelial thickness was observed in the mid-periphery.<br>-All eyes show significant epithelial remodeling during the first 6 months. All remodeling was stable without significant changes after 6 months, and there was no return to preoperative epithelial thickness.
40 eyes[13]	22.30 ± 4.62	6 months	−6.04 ± 2.18 (−2.00 to −10.00)	128.70 ± 21.91 (69 to 164)	RTVue-XR OCT (Optovue, Inc., Fremont, CA)<br>- 9 mm area	-The greatest epithelial thickness is observed in the paracentral zone (9.75%), followed by the central (8.79%,), mid-peripheral (8.12%), and peripheral zones(and 0.98%).<br>-For the midperipheral and peripheral zones, the average epithelial thickness of the flat meridian show the strongest thickening trends compared with the steep meridian.
64 eyes[14]	28.14 ± 6.38	6 months	−5.76 ± 2.01 (−1.25 to−9.88)		RTVue-XR OCT (Optovue, Inc., Fremont, CA) - 9 mm area	-The average epithelial thickness of the temporal section is significantly thicker than nasal section in paracentral ($P < 0.001$) and mid-peripheral zones ($P = 0.049$).<br>-The average epithelial thickness of superior sections is significantly thinner than that of inferior sections in paracentral, mid-peripheral, and peripheral zones (All: $P < 0.001$)

https://doi.org/10.1371/journal.pone.0294121.t005

the two groups with no statistically significant difference in factors such as preoperative MRSE, corneal ablation, optic zone diameter, and cap thickness. In addition, the effect on epithelial thickness change was further analyzed by conducting Spearman's correlation analysis separately in each group. The second limitation is that the follow-up period of 6 months was not long enough to observe any refractive regression or to conclude that corneal epithelial remodeling had finished. Corneal epithelial hyperplasia after high myopic correction is known as a myopic regression factor [10]. In this study, there was no difference in visual acuity correction at 6 months between the two groups, but further research is needed to see if myopic regression continues to be suppressed in the SMILE-xtra group, which had a relatively more stromal ablation.

In conclusion, the SMILE-xtra which performed in the relatively high-risk group of corneal ectasia based on preoperative evaluation and the relatively large corneal ablation did not show a significant difference in the pattern of corneal epithelial remodeling compared to the SMILE

group during the 6-month follow-up period. Rather, there was no significant difference in corneal stroma ablation depth, but corresponding corneal epithelial hyperplasia could be observed in SMILE-xtra with a thin RST. This study is meaningful as the first study to look at changes in the corneal epithelium in the SMILE combined with CXL using SD-OCT. Long-term studies are needed to investigate the relationship between the degree of corneal epithelial hyperplasia and myopic regression in SMILE-xtra. In addition, a comparative study with other corneal laser refractive surgeries performed concurrently with CXL is considered necessary.

Supporting information

S1 Data.
(XLSX)

Author Contributions

Conceptualization: Kook Young Kim, Sinwoo Bae, Seongjun Lee.

Data curation: Kook Young Kim, Sinwoo Bae.

Formal analysis: Kook Young Kim.

Supervision: Seongjun Lee, Yongwoo Lee.

Writing – original draft: Kook Young Kim, Yongwoo Lee.

Writing – review & editing: Kook Young Kim, Yongwoo Lee.

References

1. Reinstein DZ, Silverman RH, Sutton HF, Coleman DJ. Very high-frequency ultrasound corneal analysis identifies anatomic correlates of optical complications of lamellar refractive surgery: anatomic diagnosis in lamellar surgery. Ophthalmology. 1999; 106(3):474–82.
2. Huang D, Tang M, Shekhar R. Mathematical model of corneal surface smoothing after laser refractive surgery. American journal of ophthalmology. 2003; 135(3):267–78.
3. Brar S, Gautam M, Sute SS, Ganesh S. Refractive surgery with simultaneous collagen cross-linking for borderline corneas-a review of different techniques, their protocols and clinical outcomes. Indian Journal of Ophthalmology. 2020; 68(12):2744.
4. Sanchez-Gonzalez J-M, Rocha-de-Lossada C, Borroni D, De-Hita-Cantalejo C, Alonso-Aliste F. Prophylactic corneal crosslinking in myopic small-incision lenticule extraction-Long-term visual and refractive outcomes. Indian Journal of Ophthalmology. 2022; 70(1):73.
5. Chen X, Stojanovic A, Liu Y, Chen Y, Zhou Y, Utheim TP. Postoperative changes in corneal epithelial and stromal thickness profiles after photorefractive keratectomy in treatment of myopia. Journal of Refractive Surgery. 2015; 31(7):446–53.
6. Latifi G, Mohammadi SS, Davoodabadi M. Longitudinal epithelial thickness profile changes 18 Months after photorefractive keratectomy. Cornea. 2021; 40(4):430–9.
7. Kanellopoulos AJ, Asimellis G. Epithelial remodeling after femtosecond laser-assisted high myopic LASIK: comparison of stand-alone with LASIK combined with prophylactic high-fluence cross-linking. Cornea. 2014; 33(5):463–9.
8. Reinstein DZ, Archer TJ, Gobbe M. Lenticule thickness readout for small incision lenticule extraction compared to artemis three-dimensional very high-frequency digital ultrasound stromal measurements. Journal of Refractive Surgery. 2014; 30(5):304–9.
9. Luft N, Ring MH, Dirisamer M, Mursch-Edlmayr AS, Kreutzer TC, Pretzl J, et al. Corneal epithelial remodeling induced by small incision lenticule extraction (SMILE). Investigative Ophthalmology & Visual Science. 2016; 57(9):OCT176-OCT83. https://doi.org/10.1167/iovs.15-18879
10. Ganesh S, Brar S, Relekar KJ. Epithelial thickness profile changes following small incision refractive lenticule extraction (SMILE) for myopia and myopic astigmatism. Journal of Refractive Surgery. 2016; 32(7):473–82.

11. Ryu I-H, Kim BJ, Lee J-H, Kim SW. Comparison of corneal epithelial remodeling after femtosecond laser–assisted LASIK and small incision lenticule extraction (SMILE). Journal of refractive surgery. 2017; 33(4):250–6.

12. Kanellopoulos AJ. Comparison of corneal epithelial remodeling over 2 years in LASIK versus SMILE: a contralateral eye study. Cornea. 2019; 38(3):290–6. https://doi.org/10.1097/ICO.0000000000001821

13. Yu N, Ye Y, Chen P, Yang Y, Zhuang J, Yu K. Corneal epithelial thickness changes following SMILE for myopia with high astigmatism. Journal of Refractive Surgery. 2021; 37(4):224–30.

14. Ye Y, Chen P, Yu N, Wan L, Lan M, Zheng H, et al. Evaluation of wide corneal epithelial remodeling after small incision lenticule extraction (SMILE) with wide-field optical coherence tomography. Journal of Ophthalmology. 2022;2022.

15. Randleman JB, Woodward M, Lynn MJ, Stulting RD. Risk assessment for ectasia after corneal refractive surgery. Ophthalmology. 2008; 115(1):37–50. e4.

16. Ganesh S, Brar S. Clinical outcomes of small incision lenticule extraction with accelerated cross-linking (ReLEx SMILE Xtra) in patients with thin corneas and borderline topography. Journal of Ophthalmology. 2015;2015.

17. Schmidl D, Schmetterer L, Witkowska KJ, Unterhuber A, dos Santos VA, Kaya S, et al. Tear film thickness after treatment with artificial tears in patients with moderate dry eye disease. Cornea. 2015; 34 (4):421–6.

18. Harper C, Boulton M, Bennett D, Marcyniuk B, Jarvis-Evans J, Tullo A, et al. Diurnal variations in human corneal thickness. British Journal of Ophthalmology. 1996; 80(12):1068–72.

19. Kanellopoulos AJ. Longitudinal postoperative lasik epithelial thickness profile changes in correlation with degree of myopia correction. Journal of refractive surgery. 2014; 30(3):166.

20. Reinstein DZ, Archer TJ, Gobbe M, Silverman RH, Coleman DJ. Epithelial thickness after hyperopic LASIK: three-dimensional display with Artemis very high-frequency digital ultrasound. Journal of Refractive Surgery. 2010; 26(8):555–64.

21. Ivarsen A, Fledelius W, Hjortdal JØ. Three-year changes in epithelial and stromal thickness after PRK or LASIK for high myopia. Investigative ophthalmology & visual science. 2009; 50(5):2061–6.

22. Lohmann CP, Reischl U, Marshall J. Regression and epithelial hyperplasia after myopic photorefractive keratectomy in a human cornea. Journal of Cataract & Refractive Surgery. 1999; 25(5):712–5.

23. Spadea L, Fasciani R, Necozione S, Balestrazzi E. Role of the corneal epithelium in refractive changes following laser in situ keratomileusis for high myopia. Slack Incorporated Thorofare, NJ; 2000. p. 133–9.

24. Prakash G, Agarwal A, Mazhari AI, Chari M, Kumar DA, Kumar G, et al. Reliability and reproducibility of assessment of corneal epithelial thickness by fourier domain optical coherence tomography. Investigative Ophthalmology & Visual Science. 2012; 53(6):2580–5.

25. Yücekul B, Dick HB, Taneri S. Systematic Detection of Keratoconus in Optical Coherence Tomography: Corneal and Epithelial Thickness Maps. Journal of Cataract & Refractive Surgery. 2022:10.1097.

26. Thorsrud A, Nicolaissen B, Drolsum L. Corneal collagen crosslinking in vitro: Inhibited regeneration of human limbal epithelial cells after riboflavin–ultraviolet-A exposure. Journal of Cataract & Refractive Surgery. 2012; 38(6):1072–6.

27. Moore JE, Atkinson SD, Azar DT, Worthington J, Downes CS, Courtney DG, et al. Protection of corneal epithelial stem cells prevents ultraviolet A damage during corneal collagen cross-linking treatment for keratoconus. British Journal of Ophthalmology. 2014; 98(2):270–4.

28. Sharma A, Nottage JM, Mirchia K, Sharma R, Mohan K, Nirankari VS. Persistent corneal edema after collagen cross-linking for keratoconus. American journal of ophthalmology. 2012; 154(6):922–6. e1.

29. Kanellopoulos AJ. Collagen cross-linking in early keratoconus with riboflavin in a femtosecond laser-created pocket: initial clinical results. Journal of refractive surgery (Thorofare, NJ: 1995). 2009; 25 (11):1034–7.

30. Georgeon C, Marciano I, Cuyaubère R, Sandali O, Bouheraoua N, Borderie V. Corneal and epithelial thickness mapping: comparison of swept-source-and spectral-domain-optical coherence tomography. Journal of Ophthalmology. 2021; 2021:1–6.

31. Li WY, Hsiao C, Graham AD, Lin MC. Corneal epithelial permeability: ethnic differences between Asians and non-Asians. Contact Lens and Anterior Eye. 2013; 36(5):215–8.

32. Ivarsen A, Hjortdal J. Correction of myopic astigmatism with small incision lenticule extraction. Journal of Refractive Surgery. 2014; 30(4):240–7.

33. Sekundo W. Small incision lenticule extraction (SMILE): principles, techniques, complication management, and future concepts: Springer; 2015.

34. Shaw AJ, Collins MJ, Davis BA, Carney LG. Eyelid pressure: inferences from corneal topographic changes. Cornea. 2009; 28(2):181–8.

35. Du C, Wang J, Cui L, Shen M, Yuan Y. Vertical and horizontal corneal epithelial thickness profiles determined by ultra-high resolution optical coherence tomography. Cornea. 2012; 31(9):1036.
